# COVID-19 pandemic policing and public (non)compliant behaviour: dataset from Nigeria

**DOI:** 10.1186/s13104-023-06350-x

**Published:** 2023-05-15

**Authors:** Aliu Oladimeji Shodunke, Sodiq Abiodun Oladipupo, Sheriffdeen Olasunkanmi Adeoti, Abdulgafar Abidemi Olorede, Adebusayo Joel Alowolodu, Oriyomi Oluwasegun Seriki, Ayobami Habeeb Akindele, Isiaka Adam Folorunsho, Glory David Adebayo, Onyinye Faith Mbanefo

**Affiliations:** 1 Department of Criminology and Security Studies, University of Ilorin, Ilorin, Nigeria; 2 Department of Economics, University of Ilorin, Ilorin, Nigeria; 3 Department of Social Work, University of Ilorin, Ilorin, Nigeria; 4 Department of Psychology, University of Ibadan, Ibadan, Nigeria; 5 Department of Cooperative Economics and Management, Nnamdi Azikiwe University, Awka, Nigeria

**Keywords:** COVID-19 pandemic, Public Health, Lockdown, Disaster Management, Public Behavior, Policing, Law Enforcement, Nigeria

## Abstract

**Objectives:**

The unprecedented nature of COVID-19 pandemic lockdown order projected to contain the pandemic and the global use of the police to enforce the order has necessitated the investigation of public (non-compliant) behavior and police intervention (misconduct). Given that the phases of easing the lockdown and reopening of the economy were already underway in Nigeria in September 2020, four months post-lockdown, this period was deemed suitable to collect the data.

**Data description:**

The data consists of 30 participants’ (25 individuals and five police personnel) views regarding the reasons that exacerbated the violation and the ‘alleged’ unethical practices of police personnel while enforcing the lockdown. However, it benefits the broader scientific community in areas such as policing, disaster risk reduction, pandemic management and public administration. It is valuable in police reforms against unethical practices and gives clear policy directions to policymakers and authorities in managing future public health emergencies. Also, it is useful in understanding the public awareness about the pandemic and public (mis)trust and disposition towards the government authorities on the obedience to law and public health safety advisories to contain a pandemic.

## Objective

In January 2020, the World Health Organization (WHO) declared COVID-19 a public health emergency of global concern and a pandemic in March 2020 [2]. The pandemic’s index case was recorded in Nigeria on February 27th, 2020 [3]and heightened the preparedness and containment measures already in place before the pandemic’s outbreak. In line with the global measures, the Nigerian authorities implemented lockdown as a non-pharmaceutical policy response to contain the pandemic. The implementation resulted in a statewide/nationwide ban on inter-states movement, cessation of air traffic, closure of schools and public places, business organizations and government establishments and deployment of police personnel to implement the lockdown but with an exemption of essential workers and services [1, 4, 5]. Coupled with the fact that the majority of the Nigerian workforce are informal sector workers who do not have social security against unprecedented commercial and economic interruptions, the issues of insufficiency in relief materials gave rise to public resentment and defiance against the order [1]. Hence, it became imperative to unravel the issues of public (non)compliance and police misconduct as reported in the media and insinuated in different quarters. The data is based on extensive research on pandemic policing and public behavior in Nigeria [1] Specifically, the objectives behind the data include the following:

1. To ascertain the public’s opinion on using a lockdown to contain the pandemic.
2. To examine the factors that exacerbated non-compliance, whether economic or non-economic.
3. To (in)validate claims of police illegalities reported in the media.
4. To pinpoint the enforcement mechanism and challenges the police could face in enforcing the movement restriction.
5. To identify the public’s concerns regarding the lockdown implementation and their advice for the government and relevant authorities.
6. To assist extensively in researching pandemic policing, law enforcement, public health and public administration during an emergency.

## Data description

Qualitative data were collected to investigate the affective feeling and insights of the public regarding compliance with the lockdown directives and allegations of unethical police practices. As shown in Table 1 referenced in [8], the data consists of participants’ demography, their views on the existence of the pandemic, appropriateness of the lockdown enforced in Nigeria in March/April/May 2020, their motivation for (non)compliance, mechanisms for enforcement, police-citizens encounters, and enforcement challenges encountered. Via a semi-structured interview of participants recruited throughconvenience, referrer, venue-based, and purposive sampling techniques, the data was collected in September 2020 from 30 participants. The participants were twenty residents, five commercial tour/bus drivers who resided in Ilorin during the lockdown period and five police officers who were among the officers deployed to enforce the order. While convenience and referrer methods were used to recruit the 20 residents, venue-based and purposive techniques were used to select the tour/bus drivers and police personnel, respectively.

**Table 1:** Overview of data files/data sets

Label	Name of data file/data set	File types (file extension)	Data repository and identifier (DOI or accession number)
Data File 1	Interview Questions	Docx	Harvard Dataverse (10.7910/DVN/ZY4GKF) [8]
Data File 2	Demographic Information	Docx	Harvard Dataverse (10.7910/DVN/ZY4GKF) [8]
Data File 3	Data of Police Personnel	Docx	Harvard Dataverse (10.7910/DVN/ZY4GKF) [8]
Data File 4	Data of Members of the Public	Docx	Harvard Dataverse (10.7910/DVN/ZY4GKF) [8]
Data File 5	Data of Commercial Bus Drivers	Docx	Harvard Dataverse (10.7910/DVN/ZY4GKF) [8]
Data File 6	Data Methodology	Docx	Harvard Dataverse (10.7910/DVN/ZY4GKF) [8]

Furthermore, the data was processed using an inductive thematic analysis approach. The processing became necessary to discuss the participants’ opinions and draw inferences on the subject matter in line with the objectives of the associated study [1]. It commenced with transcription, which refers to analyzing a phenomenon or occurrence based on the transcription of recorded audio (typically spoken word) into written form [6]. The processing was necessary and was done by familiarizing with the obtained data, comprehending themes, and drawing conclusions from the transcripts of that data. The interview transcripts were re-read several times to identify recurring themes. The interviewees’ views on important topics are conveyed in explicit remarks [7]. The first step in the analytical procedure was to go over the transcript and identify frequent and distinct themes.

Upon processing and analysis, the data shows that while knowledge of happenings, hospitalization of infected individuals, the attitude of the government, and public health safety practices elicited participants’ belief in the outbreak of COVID-19 in Ilorin, Kwara State and Nigeria in general, public mistrust and institutional corruption triggered disbelief. The factors mentioned above influenced compliance as the majority of the participants obeyed the lockdown order. The reason for violation lies in insufficiency of relief materials, pre-existing economic inequalities, visitation to family, academic factors and trading of non-essential items. However, the police faced many challenges such as an inadequacy in personal protective equipment (PPE), lack of police welfare, ignorance of the public on the exempted set of people (essential workers), restiveness, and anger from the aggrieved public. Regarding police-public encounters, the data suggest that some participants either experienced or witnessed a series of police misconduct in the form of immoderate use of force, brutality, bribery and police violence which defeated the core purposes of imposing a movement restriction. Finally, the data indicate that the curfew enforcement lacked procedural justice, which entails fair and just police treatment of the public.

## Limitations

The limitations of this data rest on sample size and source of the data (location). The sample size, which is 30, is relatively small but befitting for qualitative research the associated study is based on [1]. Qualitative research entails getting behavioral insights from a small population, unlike a quantitative type that targets a large population. As small as it seems to be, the data from the sample captures relevant demographics, particularly, age, educational level and income status, which predicts public (non)compliance with the lockdown directives. In addition, the data included a very crucial population, the police, and their involvement in enforcing the lockdown. Hence, the data provides needed understanding of key issues surrounding the pandemic policing and public behavior.

Furthermore, the source of data is also a limitation. This data was collected from different communities in a single location, Ilorin, Kwara State, Nigeria, thus, raising concern about the generalizability of the findings to the rest of Nigeria. However, it is noteworthy that the choice of Ilorin city stemmed from its strategic position, socio-economic peculiarities and geography. Apart from its accommodation of a handful of natives of the major tribes, the city connects directly to the country’s commercial hub, Lagos (306 km distance) and the capital, Abuja (500 km distance). Also, it maintains travel routes with major cities and transport hubs across the Northern and Southern of the country. There are a cheap affordability of transport means within Kwara State. Thus, these peculiarities of Ilorin within Kwara State and with other states make the data collected useful and reliable as they could facilitate the violation of lockdown (travel) directives, in which this data is based on.

## Data Availability

The data described in this Data note can be freely and openly accessed on [Harvard Dataverse] under [10.7910/DVN/ZY4GKF] Please see Table 1 and references [8] for details and links to the data.

## Abbreviations

WHO: World Health Organization
COVID-19: Coronavirus Disease 2019
PPE: Personal Protective Equipment
